# Plaque stage folliculotropic mycosis fungoides: histopathologic features and prognostic factors in a series of 40 patients

**DOI:** 10.1111/cup.13615

**Published:** 2019-12-09

**Authors:** Suzanne van Santen, Patty M. Jansen, Koen D. Quint, Maarten H. Vermeer, Rein Willemze

**Affiliations:** ^1^ Department of Dermatology Leiden University Medical Center Leiden The Netherlands; ^2^ Department of Clinical Pathology Leiden University Medical Center Leiden The Netherlands

**Keywords:** folliculotropic mycosis fungoides, histopathology, prognosis, risk factor

## Abstract

**Background:**

Folliculotropic mycosis fungoides (FMF) is a distinct variant of mycosis fungoides. Recent studies recognized indolent and aggressive subgroups of FMF, but there is controversy how patients presenting with plaques should be classified. The present study describes the histopathologic features of 40 FMF plaques. The aim of the study was to identify risk factors for disease progression and poor outcome in this group.

**Methods:**

Clinical, histopathological, and immunophenotypical data from 40 patients with plaque stage FMF were reviewed and analysed for risk factors for disease progression and survival.

**Results:**

After a median follow‐up of 80 months, disease progression occurred in 20 of 40 patients. Percentage of atypical cells, cell size, percentage of Ki‐67+ cells, and co‐existent interfollicular epidermotropism, but not the extent of perifollicular infiltrates, were associated with disease progression and reduced survival, while extensive follicular mucinosis was associated with increased survival.

**Conclusions:**

This study underlines that FMF patients presenting with plaques represent a heterogeneous group and that a subgroup of these patients may have an indolent clinical course. It further shows that histological examination is a valuable tool to differentiate between indolent and aggressive disease.

## 1 INTRODUCTION

Folliculotropic mycosis fungoides (FMF) is recognized as a distinct variant of mycosis fungoides (MF). FMF is histopathologically characterized by the presence of folliculotropic infiltrates consisting of atypical, T‐cells, that often spare the epidermis.^1^, ^2^, ^3^ Clinically, skin lesions are preferentially located in the head and neck region and are frequently accompanied by local alopecia, and may include (grouped) follicular papules, acneiform and cystic lesions, plaques and tumors and in some patients keratosis pilaris‐like skin lesions, which are located preferentially on the extremities or trunk.^1^, ^2^, ^4^, ^5^, ^6^, ^7^, ^8^, ^9^, ^10^, ^11^, ^12^, ^13^ Previous studies emphasized that FMF patients have a worse prognosis when compared to classic MF patients and should be treated more aggressively.^1^, ^2^ However, more recent studies reported that not all patients with FMF have an unfavorable prognosis and suggested that distinction should be made between an indolent (early stage) and an aggressive group (advanced stage) of FMF.^5^, ^7^, ^11^ Patients presenting with only follicle‐based patches, acneiform, or keratosis pilaris‐like lesions have early‐stage disease (stages IA‐IIA), run an indolent clinical course and have an excellent prognosis, while patients presenting with nodules, tumors, erythroderma, and/or extracutaneous disease have advanced FMF (stages IIB‐IV) and usually show a much more aggressive clinical course.^5^ It is however uncertain how patients presenting with plaques should be categorized. In a study of Hodak et al, all patients with infiltrated plaques were upstaged and considered to have tumor‐stage disease (stage IIB).^7^ In contrast, studies of our own group showed that a subset of patients presenting with plaques may have a favorable clinical course. Using histopathological criteria, distinction was made between cases with early plaque‐stage FMF and advanced plaque‐stage FMF. It was found that patients with early plaque‐stage FMF had the same indolent clinical behavior as patients with patch‐stage FMF, whereas patients with advanced plaque‐stage FMF had the same clinical behavior and prognosis as patients with tumor‐stage FMF.^5^ Histopathologic classification as either early or advanced plaque‐stage disease was based on a rough estimate of the extent and cellular composition of the perifollicular infiltrates in a single hematoxylin and eosin (H&E)‐stained section, but predefined criteria were lacking.^5^ In the present study, clinical, histopathological, and immunophenotypical characteristics of 40 patients with plaque stage FMF at diagnosis were evaluated. The aim of this study was to describe histopathological characteristics of plaque stage FMF and assess parameters associated with disease progression and survival.

### 1.1 Patients and methods

The database of the Dutch Cutaneous Lymphoma Group was searched for patients with FMF, who met the following criteria: the clinical presence of plaques without concurrent tumors or extracutaneous disease at the time of diagnosis, availability of clinical illustrations, and availability of relevant immunostainings or tissue blocks to perform additional stains. Plaques were defined clinically as palpable, mostly elevated skin lesions with a diameter > 1 cm.^14^ In order to study the risk of disease progression or death of lymphoma, only patients with a follow‐up period of at least 60 months or death before that time were selected. The total study group included 40 patients with plaque stage FMF. In all cases, the diagnosis of FMF met the criteria of the WHO‐EORTC classification and was confirmed by an expert panel of dermatologists and pathologists of the Dutch Cutaneous Lymphoma Group.^15^ Clinical and follow‐up data were retrieved from the Dutch Cutaneous Lymphoma Registry and from medical records. The date of first diagnostic biopsy was considered the time of diagnosis. Disease progression was defined as the development of skin tumors (stage IIB) or extracutaneous disease (stage IV) or death due to lymphoma. The study was performed in accordance with the Declaration of Helsinki and in accordance with our in‐house Biobank protocol, as approved by our Medical Ethics Committee.

### 1.2 Histopathologic parameters

Histopathologic sections from the initial diagnostic skin biopsy including routinely stained H&E sections and a panel of immunostains were reviewed by three of the authors (SvS, PJ, and RW) blinded to the clinical and follow‐up data. The following parameters were evaluated: extent of neoplastic infiltrate (sparse (1) or (2) prominent perifollicular and perivascular infiltrates confined to the perifollicular area; (3) confluent perifollicular and interfollicular infiltrates; (4) completely diffuse infiltrates), the percentage of atypical cells in the infiltrate (<10%; 11%‐25%; >25%), size of the neoplastic T‐cells (predominantly small/medium or predominantly medium/large), the percentage of blast cells (<10%; 11%‐25%; >25%), degree of folliculotropism (mild, moderate, or extensive), presence or absence of interfollicular epidermotropism, presence, or absence of syringotropism, presence of follicular mucinosis (no or minimal vs prominent) and presence or absence of eosinophils and neutrophils (no or few vs prominent). Immunohistochemical sections, routinely stained for CD2, CD3, CD4, CD5, CD8, CD20, CD30, CD68, Ki‐67, and cytotoxic proteins (TIA‐1, granzyme B) were reviewed to determine the phenotype of neoplastic cells and the extent and composition of admixed inflammatory infiltrate. The presence of Ki‐67+ cells was scored as more or less than 10%.

### 1.3 Statistical analysis

All statistical analyses were performed using the SPSS statistical software (IBM SPSS Statistics 23). To determine statistically significant clinical or histopathological differences between patients with disease progression (P‐FMF) and patients without disease progression (NP‐FMF) independent samples *t* tests and χ² tests were used. *P*‐values <0.05 were considered statistically significant. Disease‐specific survival (DSS) was calculated from the date of diagnostic biopsy until death as result of lymphoma or date of last follow‐up. Overall survival (OS) was calculated from the date of diagnosis until patient's death from any cause or date of last follow‐up. Progression‐free survival (PFS) was calculated from the date of diagnosis until time of disease progression or death. Survival curves were calculated using Kaplan‐Meier analyses and log‐rank tests were used for comparison between survival curves. Using a Cox proportional hazards model, univariate analysis of age, and all histopathologic parameters was performed. Factors significant in univariate analysis were included in a multivariate analysis model. In both models, *P*‐values below 0.05 were considered significant.

## 2 RESULTS

### 2.1 Clinical characteristics

The study included 29 males and 11 females (ratio: 2.6) with a median age at diagnosis of 56 years (range: 19‐82 years). Distinction was made between patients with disease progression (P‐FMF) and patients without disease progression for at least 5 years after diagnosis (NP‐FMF). In the P‐FMF group, the median time from diagnosis to disease progression was 30 months (range, 3‐136 months) and occurred within 5 years after diagnosis in 17 of 20 patients and after 61, 66, and 136 months in the other three patients. The main clinical characteristics of these two groups are presented in Table 1.

**Table 1:** Clinical characteristics of 40 patients with plaque stage folliculotropic mycosis fungoides

	Total group (n = 40)	NP‐FMF (n = 20)	P‐FMF (n = 20)	*P*‐value
Median age at diagnosis (range; months)	56 (19‐82)	51 (19‐68)	62 (29‐82)	<0.01
Male‐female (ratio)	29‐11 (2.6)	15‐5 (3.0)	14‐6 (2.3)	0.72
Median time from first skin lesion to diagnosis (months)	24 (3‐300)	10 (2‐360)	36 (3‐300)	0.19
Extent of skin lesions				0.11
Solitary	2 (5%)	2 (10%)	0	
Localized	2 (5%)	2 (10%)	0	
Generalized	36 (90%)	16 (80%)	20 (100%)	
Predominant head/neck involvement	32 (80%)	16 (80%)	16 (80%)	1.0
Pruritus				0.34
Yes	30 (75%)	13 (65%)	17 (85%)	
No	8 (20%)	5 (25%)	3 (15%)	
Unknown	2 (5%)	2 (10%)	—	
Type of initial treatment				0.61
Topical steroids	4 (10%)	3 (15%)	1 (5%)	
Narrow‐band UVB	2 (5%)	0 (0%)	2 (10%)	
PUVA	16 (40%)	9 (45%)	7 (35%)	
PUVA + local radiotherapy	6 (15%)	2 (10%)	4 (20%)	
Local radiotherapy	6 (15%)	3 (15%)	3 (15%)	
Total skin radiotherapy	6 (15%)	3 (15%)	3(15%)	
Result initial treatment				0.03
Complete remission	15 (37%)	12 (60%)	3 (15%)	
Partial remission	13 (32%)	3 (15%)	10 (50%)	
Stable disease	10 (25%)	4 (20%)	6 (30%)	
Progressive disease	2 (5%)	1 (5%)	1 (5%)	
Median duration follow‐up (range) (months)	80 (6‐320)	104 (38‐320)	59 (6‐241)	0.05
Status at last follow‐up				<0.01
Alive without disease	7 (17%)	7 (35%)	0 (0%)	
Alive with ongoing disease	14 (35%)	10 (50%)	4 (20%)	
Died of lymphoma	15 (37%)	0 (0%)	15 (75%)	
Died of unrelated disease	4 (10%)	3 (15%)	1 (5%)	
Disease‐specific survival at 5/10 years	72%/57%	100%/100%	45%/24%	<0.01
Overall survival at 5/10 years	68%/54%	90%/90%	45%/24%	<0.01

Abbreviations: NP‐FMF: non‐progressive folliculotropic mycosis fungoides; P‐FMF, progressive folliculotropic mycosis fungoides; PUVA: psoralen plus ultraviolet A therapy; UVB: ultraviolet B therapy.

The median age at diagnosis of patients in the P‐FMF group was higher than in the NP‐FMF group (62 vs 51 years, respectively, *P* = 0.004). No significant differences between the P‐FMF group and the NP‐FMF group were found in the extent of skin lesions, the predominant involvement of the head and neck region, the presence of pruritus, and the initial type of treatment. The number of patients achieving complete remission upon initial treatment was higher in the NP‐FMF group than in the P‐FMF group (60% vs 15%, respectively, *P* = 0.003), but overall response rates were comparable (75% vs 65%, respectively).

Follow‐up data of the NP‐FMF group showed sustained complete remission in eight of 20 patients, while 12 patients had relapsing skin disease without progression beyond plaque stage disease. In the P‐FMF group, five of 20 patients developed only skin tumors (stage IIB), whereas 15 patients developed extracutaneous disease with or without skin tumors (stage IV). After a median follow‐up of 59 months, four patients in this P‐FMF group were alive with ongoing disease, 15 patients died of lymphoma and one died of unrelated disease. The 5‐year DSS and OS in the P‐FMF group were both 45%, compared to 100% and 89%, respectively, in the NP‐FMF group.

### 2.2 Histopathology

The main histopathological findings in these 40 patients with plaque stage FMF are summarized in Table 2. In 35 of 40 cases, biopsies were obtained from skin lesions in the head and neck area. In two cases of the NP‐FMF group and three cases of the P‐FMF group, skin lesions on the back (four cases) or arm (one case) were biopsied.

**Table 2:** Histopathologic characteristics of 40 patients with plaque stage folliculotropic mycosis fungoides

	Total group (n = 40)	NP‐FMF (n = 20)	P‐FMF (n = 20)	*P*‐value
Location of skin biopsy				0.63
Head and neck area	35 (88%)	18 (90%)	17 (85%)	
Trunk or extremities	5 (12%)	2 (10%)	3 (15%)	
Extent of dermal infiltrate				0.52
1. sparse perifollicular infiltrate	4 (10%)	3 (15%)	1 (5%)	
2. prominent perifollicular infiltrate	14 (35%)	8 (40%)	6 (30%)	
3. confluent peri−/intrafollicular infiltrates	18 (45%)	7 (35%)	11 (55%)	
4. completely diffuse infiltrate	4 (10%)	2 (10%)	2 (10%)	
Folliculotropism				0.05
Mild infiltration	6 (15%)	5 (25%)	1 (5%)	
Moderate infiltration	17 (42%)	10 (50%)	7 (35%)	
Extensive infiltration	17 (42%)	5 (25%)	12 (60%)	
Follicular mucinosis				0.01
No or focal spots	20 (50%)	6 (30%)	14 (70%)	
Moderate to extensive	20 (50%)	14 (70%)	6 (30%)	
Interfollicular epidermotropism^a^				0.01
Absent	26 (68%)	16 (88%)	10 (50%)	
Present	12 (32%)	2 (12%)	10 (50%)	
Syringotropism				0.68
Absent	33 (83%)	17 (85%)	16 (80%)	
Present	7 (17%)	3 (15%)	4 (20%)	
Percentage atypical cells^b^				0.02
<10%	7 (17%)	5 (25%)	2 (10%)	
10%‐25%	12 (30%)	9 (45%)	3 (15%)	
>25%	21 (53%)	6 (30%)	15 (75%)	
Size of atypical cells				<0.01
Predominantly small‐medium	33 (83%)	20 (100%)	13 (65%)	
Predominantly medium‐large	7 (17%)	0 (0%)	7 (35%)	
Percentage of blast cells^c^				0.15
Absent or few <10%	34 (85%)	19 (95%)	15 (75%)	
10%–25%	3 (7%)	1 (5%)	2 (10%)	
>25% (blastic transformation)	3 (7%)	0 (0%)	3 (15%)	
Percentage of CD30+ cells				0.21
<10%	33 (83%)	18 (90%)	15 (75%)	
>10%	7 (17%)	2 (10%)	5 (25%)	
Ki‐67				<0.01
<10%	29 (73%)	19 (95%)	10 (50%)	
>10%	11 (28%)	1 (5%)	10 (50%)	
Reactive CD8+ cells^d^				0.42
Absent or few <10%	16 (42%)	6 (32%)	10 (53%)	
Moderate 10%–25%	19 (50%)	11 (58%)	8 (42%)	
Many >25%	3 (8%)	2 (11%)	1 (5%)	
Eosinophils				0.29
Absent or few	29 (72%)	13 (65%)	16 (80%)	
Prominent	11 (28%)	7 (35%)	4 (20%)	

Abbreviations: NP‐FMF, non‐progressive folliculotropic mycosis fungoides; P‐FMF, progressive folliculotropic mycosis fungoides.

a In two cases evaluation not possible because of ulceration.

b Percentage of atypical cells >25% was used as a prognostic factor for statistical analysis.

c Percentage of blast cells >10% was used as a prognostic factor for statistical analysis.

d Two cases with CD8+ tumor cells were excluded.

**Figure 1:**
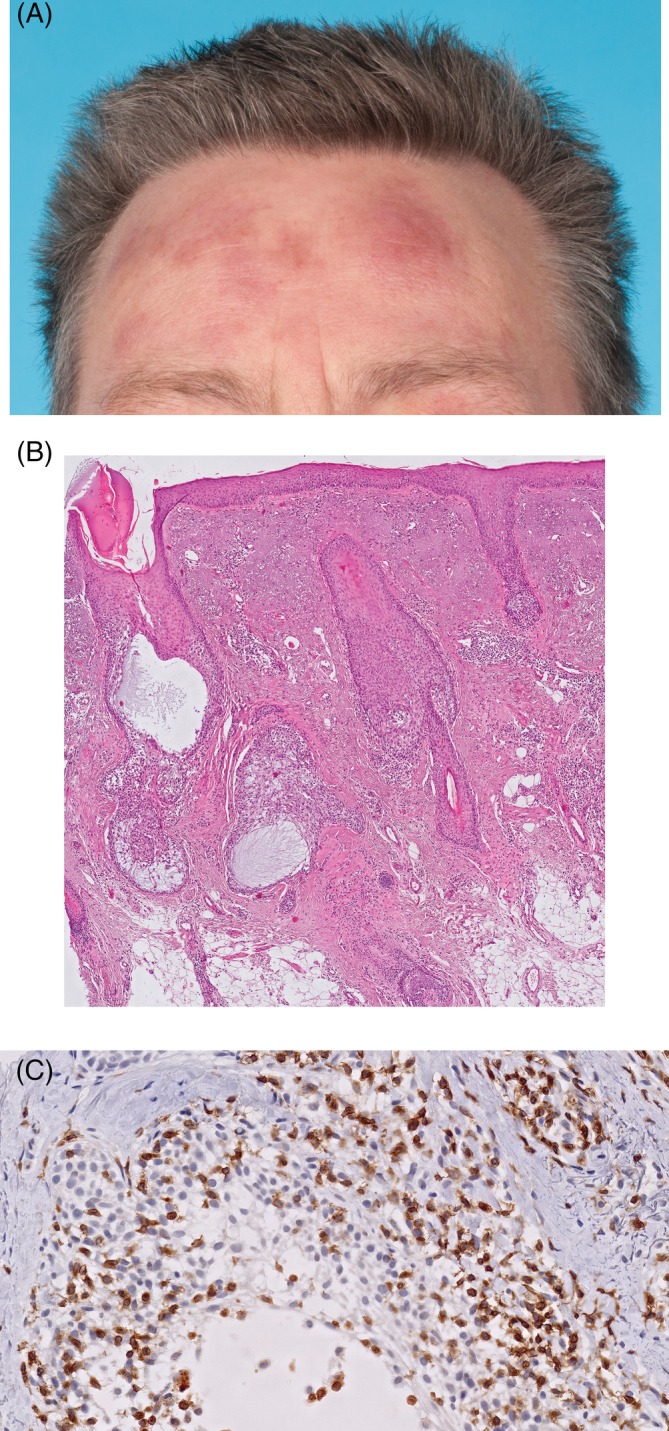
Plaques on a forehead (A). Histopathologic examination shows sparse intrafollicular and perifollicular infiltrates and extensive follicular mucinosis, which may explain the induration of the skin lesions (B, hematoxylin and eosin [H&E], ×40). Folliculotropic infiltrate shows positive staining for CD3 (C, original magnification, ×400)

**Figure 2:**
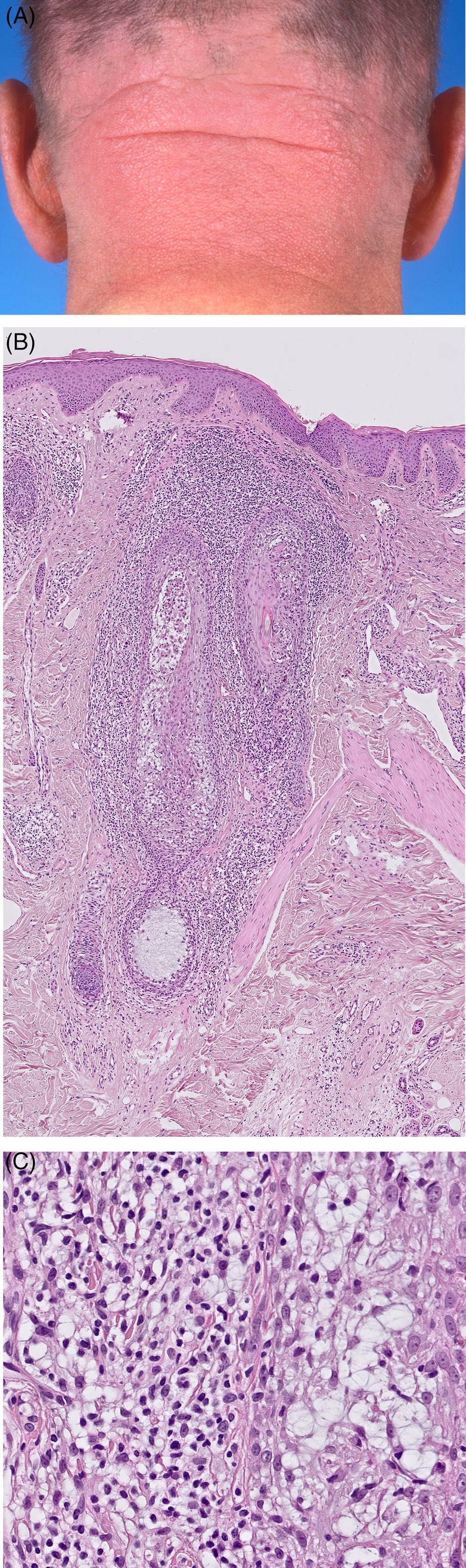
Plaque in the neck with follicular accentuation (A, hematoxylin and eosin [H&E], ×40); Prominent perifollicular infiltrates. B, Detail of the infiltrate shows infiltration by small atypical T‐cells (C, H&E, ×400)

**Figure 3:**
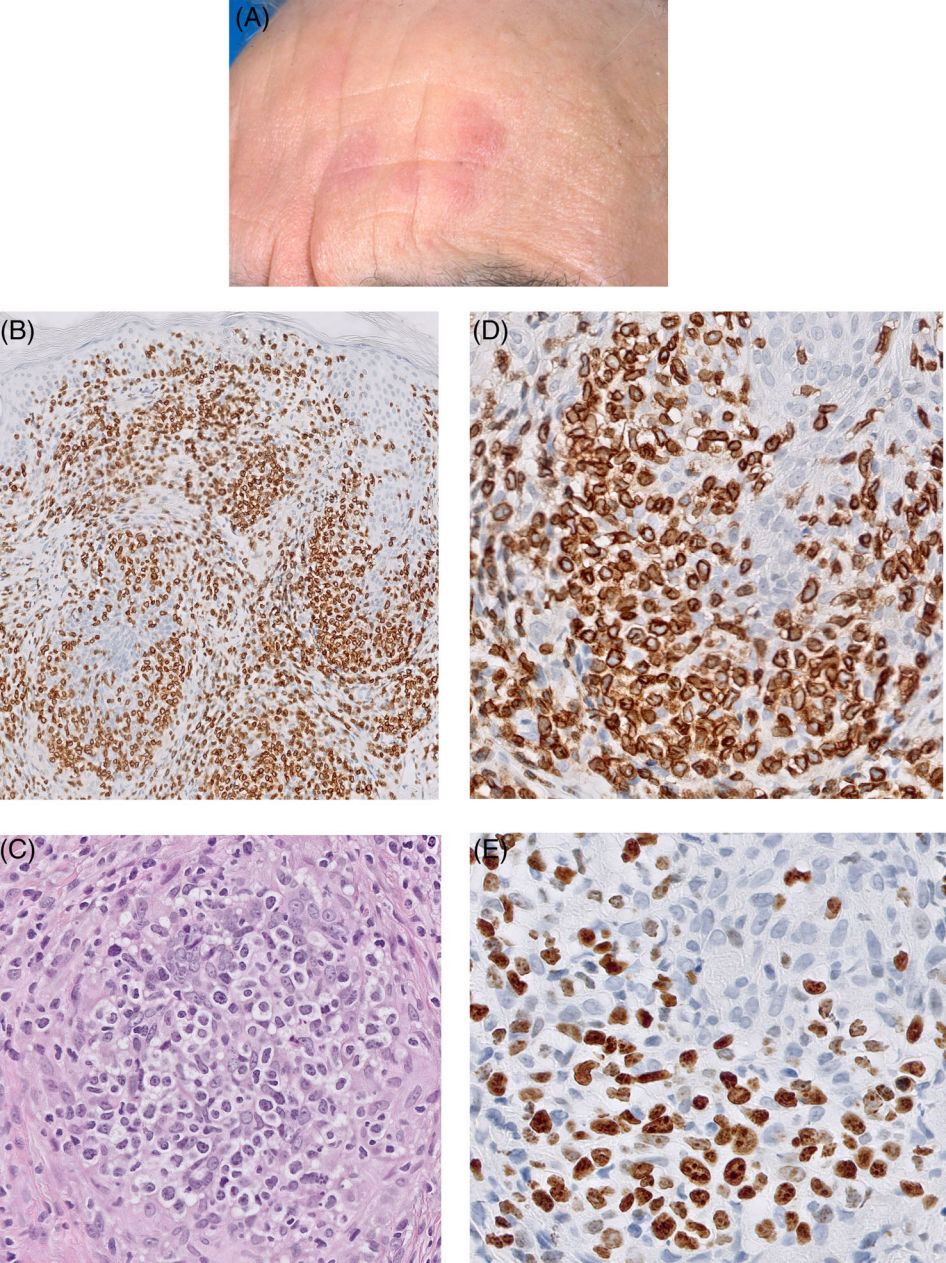
Plaques on a forehead (A). CD3 staining shows confluent perifollicular and perivascular infiltrates throughout the dermis (B, original magnification, ×40). Detail of infiltrate showing infiltration of follicular epithelium by large atypical lymphocytes (C, hematoxylin and eosin [H&E], ×400). Atypical T‐cells express CD3 (D, original magnification, ×400) and Ki‐67 staining shows a high proliferation rate (E, original magnification, ×400)

**Figure 4:**
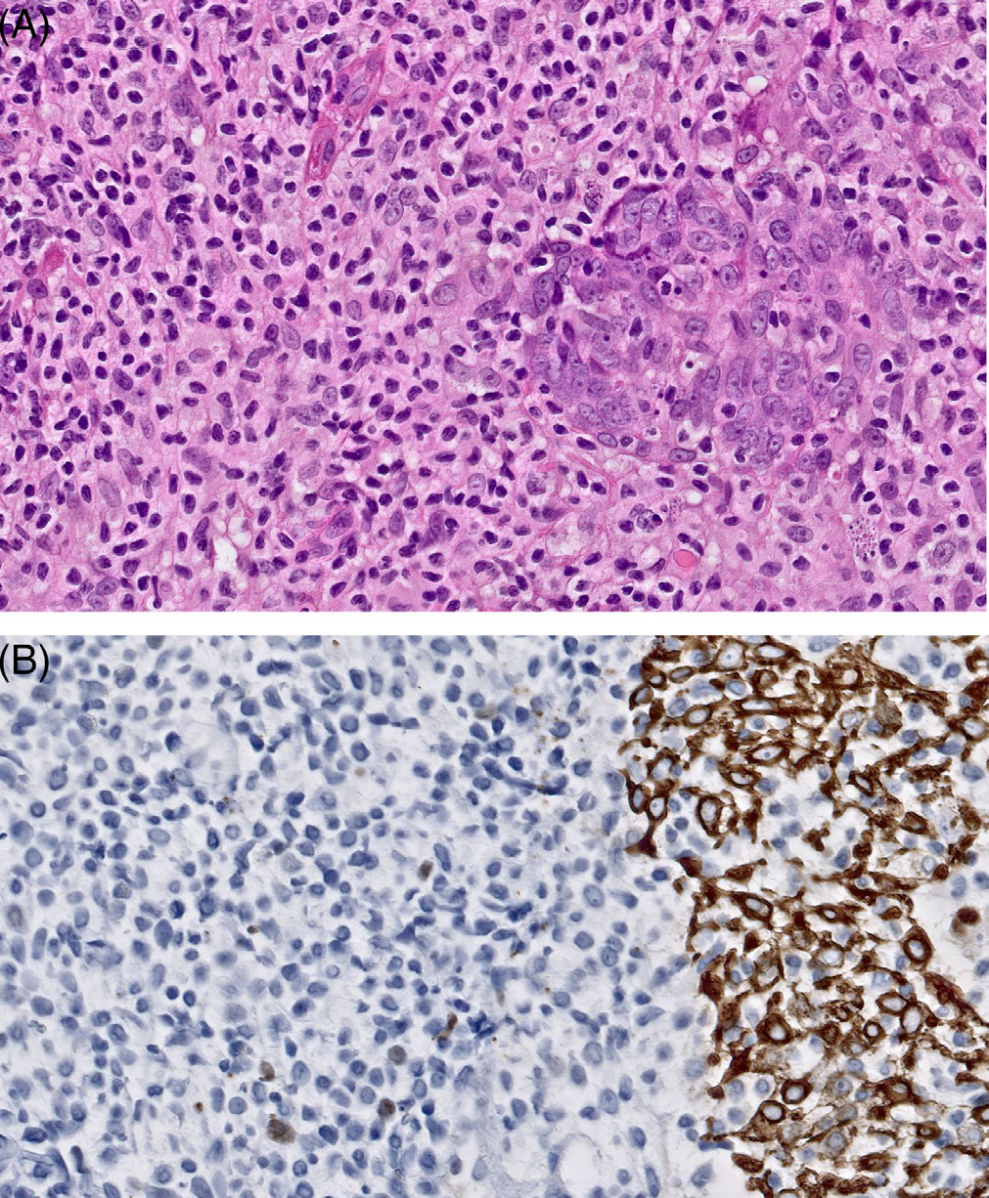
Detail of diffuse dermal infiltrate showing a dense infiltrate of small atypical T‐cells infiltrating follicular epithelium (A, hematoxylin and eosin [H&E], × 400); Keratin staining highlights remnants of follicular structures (B, original magnification, ×400)

Twenty cases showed sparse (n = 4) or prominent (n = 14) infiltrates confined to the perifollicular area **(**Figures 1 and 2 **)**. Confluent perifollicular and perivascular infiltrates or completely diffuse infiltrates were observed in the other 22 cases, and were more common in the P‐FMF group **(**Figures 3 and 4 **).** Infiltration of the follicular epithelium by atypical lymphocytes was mild to moderate in 23 cases and extensive in 17 cases, including five of 20 (25%) in the NP‐FMF group and 12 of 20 (60%) in the P‐FMF group.

**Figure 5:**
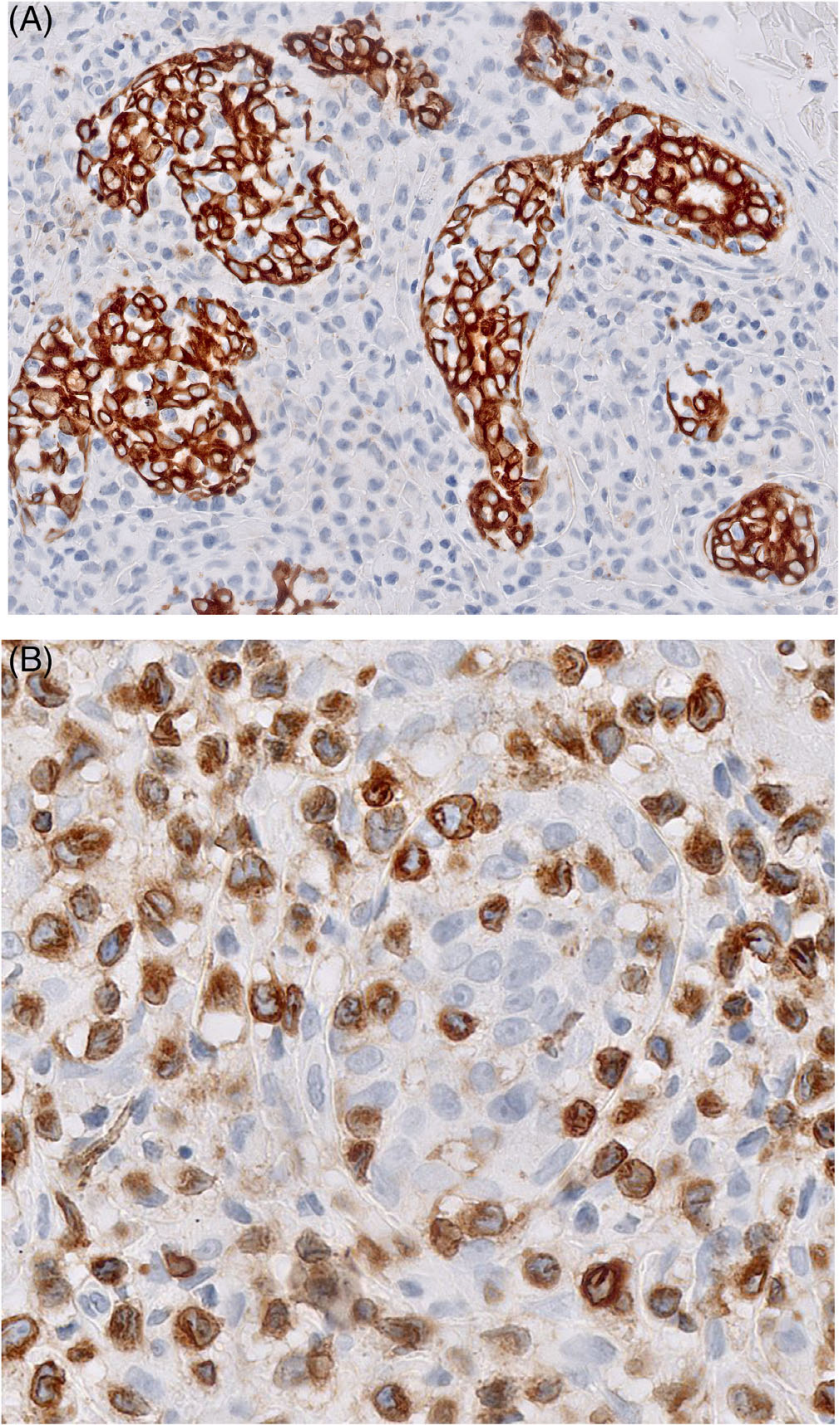
Keratin staining shows infiltration of large atypical lymphocytes into sweat glands (syringotropism) (A, original magnification, ×400); Expression of CD3 by syringotropic T‐cells (B, original magnification, ×400)

Follicular mucinosis was prominent in 14 of 20 cases (70%) in the NP‐FMF group, compared to six of 20 (30%) P‐FMF cases (*P* = 0.01), but was absent or only focally present in the other 20 cases. Coexistent infiltration of the interfollicular epidermis by atypical T‐cells was observed in 12 of 38 evaluable cases (31%), and was much more common in the P‐FMF group than in the NP‐FMF group (50% vs 11%, respectively *P* = 0.01)). Epidermotropism was mild in six cases and prominent in another six cases, but was always less pronounced than coexistent folliculotropism. Syringotropism was seen in seven of 40 cases, without any difference between the two subgroups **(**Figure 5 **)**.

Percentages of atypical cells, including cells with hyperchromatic and cerebriform nuclei and blast cells, in the dermal infiltrates were less than 10% in seven cases, 10% to 25% in 12 cases and more than 25% in 21 cases. Percentages above 25% were more frequent in the P‐FMF than in the NP‐FMF group (75% vs 30%, respectively; *P* = 0.02). In 33 of 40 cases (83%), these atypical cells were predominantly small to medium‐sized **(**Figures 2B and 4A**)**. In the other seven cases, all seven belonging to the P‐FMF group, they were predominantly medium‐sized to large **(**Figure 3C**)**. Blast cells were either few or absent in 34 of 40 cases (85%). In six cases, they made up more than 10% of the T‐cell infiltrate, but only three cases had percentages more than 25% and were considered to show large cell transformation.^16^ The neoplastic cells had a CD3+, CD4+, CD8‐ T‐cell phenotype in 38 cases and a CD3+, CD4‐, CD8+ T‐cell phenotype in two cases. Expression of CD30 by more than 10% of the neoplastic T‐cells was observed in two of 20 cases (10%) in the NP‐FMF group and five of 20 cases (25%) in the P‐FMF group. Percentages of more than 10% Ki‐67 positive cells were detected in 11 of 40 cases and were much more common in the P‐FMF group than in the NP‐FMF group (50% and 5%, respectively *P* = <0.01) **(**Figure 3E**)**. Dermal infiltrates contained variable numbers of admixed inflammatory cells, but comparison of the two subgroups of plaque stage FMF showed no significant differences. Percentages of admixed CD8+ T‐cells varied between 10% to more than 25% in 20 cases with a CD4+ T‐cell phenotype, while percentages lower than 10% were found in 18 cases. Within the follicular epithelium CD8+ T‐cells were generally few or absent. CD79a showed clusters of small B‐cells in 26 of 40 cases, with formation of reactive germinal centers in two cases. Considerable numbers of eosinophils were observed in 11 of 40 cases (clusters of) plasma cells in 11 cases, while neutrophils were generally absent. CD68 staining revealed considerable numbers of histiocytes in most cases, while variable numbers of multinucleated giant cells were observed in 12 of 40 cases.

### 2.3 Prognostic factors

Factors that significantly correlated with both disease progression and reduced survival included the presence of more than 25% atypical cells in dermal infiltrates, the size of neoplastic T‐cells, the presence of coexistent interfollicular epidermotropism and presence of more than 10% Ki‐67 positive cells. Presence of more than 10% blast cells was associated increased disease progression. In contrast, presence of prominent follicular mucinosis was associated with reduced disease progression and increased survival (Table 3). The extent of perifollicular infiltrates, which had been used in our previous study as one of the parameters to differentiate between early and advanced plaque stage disease,^5^ had no effect on prognosis and disease progression. Other factors that did not correlate with disease progression or survival were extent of folliculotropism, presence or absence of syringotropism, presence, or absence of many eosinophils and percentages of admixed reactive CD8+ T‐cells, CD30+ T‐cells, CD79a+ B‐cells, plasma cells, and CD68+ histiocytes. In multivariate analysis, size of neoplastic T‐cells was independently associated with reduced PFS, whereas the presence of >10% Ki‐67 positive cells was associated with reduced survival.

**Table 3:** Univariate and multivariate analysis of relevant histopathologic features in plaque stage folliculotropic mycosis fungoides

		DSS				OS				PFS			
	N	Univariate analysis		Multivariate analysis		Univariate analysis		Multivariate analysis		Univariate analysis		Multivariate analysis	
	40	HR (95%CI)	*P*‐value	HR (95%CI)	*P*‐value	HR (95%CI)	*P*‐value	HR (95%CI)	*P*‐value	HR (95%CI)	*P*‐value	HR (95%CI)	*P*‐value
Age at diagnosis			**0.004**		0.11		**0.001**		**0.04**		**0.001**		0.10
≤60 years	**26**	1				1				1			
>60 years	**14**	4.7 (1.6‐13.4)				5.2 (2.0‐13.4)				5.8 (2.1‐15.8)			
Extent of infiltrate			0.79		‐		0.69		‐		0.51		‐
Minimal perifollicular	**4**												
Prominent perifollicular	**14**												
Extensive perifollicular/confluent	**18**												
Completely diffuse	**4**												
Folliculotropism			0.50		‐		0.12		‐		0.04		‐
Mild	**6**										Ref		
Moderate	**17**										0.36		
Extensive	**17**										0.06		
**F**ollicular mucinosis			**0.04**		0.61		**0.009**		0.96		**0.02**		0.75
No or focal spots	**20**	1				1				1			
Moderate to extensive (lakes)	**20**	0.30 (0.10‐0.94)				0.25 (0.9‐0.71)				0.31 (0.12‐0.81)			
Interfollicular epidermotropism			**0.003**		0.33		**0.007**		0.56		**0.005**		0.34
Absent	**26**	1				1				1			
Present	**12**	4.9 (1.7‐13.8)				3.5 (1.4‐8.7)				3.6 (1.5‐8.8)			
Percentage atypical cells			**0.005**		0.28		**0.005**		0.48		**0.006**		0.60
<25%	**19**	1				1				1			
>25%	**21**	8.6 (1.9‐38.3)				5.0 (1.6‐15.2)				4.2 (1.5‐11.7)			
**Cell size**			**0.008**		0.23		**0.006**		0.37		**<0.001**		**0.03**
Small‐medium	**33**	1				1				1		1	
Medium‐large	**7**	4.4 (1.5‐13.0)				4.0 (1.5‐10.7)				9.5 (3.2‐27.6)		4.3 (1.1‐16.3)	
Percentage of blast cells			0.38		‐		0.08		‐		**0.01**		0.95
<10%	**34**									1			
>10%	**6**									3.7 (1.3‐11.2)			
Percentage of Ki‐67+ cells			**<0.001**		**0.02**		**<0.001**		**0.03**		**<0.001**		0.27
<10%	**29**	1		1		1		1		1			
>10%	**11**	7.7 (2.7‐22.3)		5.3 (1.3‐21.2)		7.3 (2.7‐19.7)		4.2 (1.2‐14.7)		5.3 (2.1‐13.0)			

*Note*: Bold and underlined values were considered significant, as p<0.05 was considered significant.

Abbreviations: CI, confidence interval; DSS, disease‐specific survival; HR: hazard ratio; N: number; OS, overall survival; PFS: progression‐free survival.

## 3 DISCUSSION

The present study describes the histopathologic features of 40 FMF plaques biopsied at diagnosis. The aim of this study was to identify risk factors for disease progression and poor outcome in this group. After a median follow‐up of 80 months, disease progression occurred in 20 of 40 patients. Histopathologic evaluation revealed that the presence of more than 25% atypical cells in dermal infiltrates, size of neoplastic cells, the presence of more than 10% blast cells, presence of interfollicular epidermotropism, and presence of more than 10% Ki‐67 positive cells were associated with decreased DSS, OS and/or PFS. Prominent follicular mucinosis was associated with increased survival and reduced risk of disease progression.

In a recent study of Hodak et al, extent and depth of perifollicular infiltrates were significantly greater in advanced‐stage FMF than in early‐stage FMF. In addition, eosinophils and plasma cells in dermal infiltrates as well as pruritus were significantly more common in advanced‐stage FMF.^7^ However, in that study early‐stage FMF included follicle‐based patches and acneiform and keratosis pilaris‐like lesions that mainly involved trunk and extremities, while advanced‐stage FMF did not only include tumors, but also infiltrated plaques that preferentially involved the head and neck region. It should be noted that our study included only plaques and that in 80% of cases biopsies had been taken from the most infiltrated lesions in the head and neck area. This might explain why extent and depth of perifollicular infiltrates as well as the other abovementioned risk factors were not different between the two groups in the present study.

The observation that co‐existent interfollicular epidermotropism is a risk factor for disease progression and reduced survival was unexpected and is at present unexplained. In 11 of 12 cases, with co‐existent epidermotropism skin biopsies were obtained from characteristic FMF lesions in the face and none of these patients had concurrent skin lesions typical of classic MF.

Previous studies in classic MF showed that low numbers of admixed CD8+ T‐cells are associated with disease progression and an inferior prognosis.^17^, ^18^, ^19^ In the present study, such an association was not found. Notably, number of reactive CD8+ T‐cells infiltrating into the follicular epithelium was very low, similar to the few or absent reactive CD8+ T‐cells in the epidermis of early‐stage classic MF.

Furthermore, we found that age at diagnosis and lack of complete response upon initial treatment were clinical prognostic factors associated with disease progression in plaque‐stage FMF. Age is a well‐reported prognostic factor in both classic MF and FMF, while complete response to initial treatment has been reported as a favorable prognostic factor in classic MF.^5^, ^6^, ^20^, ^21^, ^22^, ^23^

In conclusion, the results of the present study show that FMF patients presenting with plaques represent a heterogeneous group and that a subgroup of these patients may have an indolent clinical course. Our results argue against the suggestion that all cases of FMF with plaques should be considered to have tumor‐stage disease. They further show that histopathological examination is a valuable tool to differentiate between indolent and aggressive disease.

## CONFLICT OF INTEREST

The authors declare no potential conflict of interest.
